# CDKN2A Copy Number Loss Is an Independent Prognostic Factor in HPV-Negative Head and Neck Squamous Cell Carcinoma

**DOI:** 10.3389/fonc.2018.00095

**Published:** 2018-04-04

**Authors:** William S. Chen, Ranjit S. Bindra, Allen Mo, Thomas Hayman, Zain Husain, Joseph N. Contessa, Stephen G. Gaffney, Jeffrey P. Townsend, James B. Yu

**Affiliations:** ^1^Yale School of Medicine, New Haven, CT, United States; ^2^Department of Therapeutic Radiology, Yale School of Medicine, New Haven, CT, United States; ^3^Yale Cancer Center, Yale School of Medicine, New Haven, CT, United States; ^4^University of Connecticut School of Medicine, Farmington, CT, United States; ^5^Department of Biostatistics, Yale School of Public Health, New Haven, CT, United States; ^6^Cancer Outcomes, Public Policy, and Effectiveness Research (COPPER) Center at Yale, Yale School of Medicine, New Haven, CT, United States

**Keywords:** CDKN2A, head and neck neoplasms, prognostic biomarkers, genomics and genetics, outcomes assessment

## Abstract

**Background:**

HPV infection is associated with high p16 expression and good prognosis in head and neck squamous cell carcinomas (HNSCCs). Analysis of CDKN2A, the gene encoding p16, may further elucidate the association between p16 expression and prognosis. We sought to determine whether CDKN2A copy number loss was associated with poor survival in HPV-negative HNSCCs.

**Methods:**

The Cancer Genome Atlas HNSCC clinical and genomic data were obtained and integrated. Patients <80 years old with a primary tumor in the oral cavity, oropharynx, hypopharynx, or larynx were included. Stratifying by copy number loss status, CDKN2A mRNA and p16 protein expression levels were examined and overall survival (OS) and disease-free survival (DFS) were evaluated.

**Results:**

401 patients with HPV-negative HNSCC were identified. 146 patients demonstrated CDKN2A copy number loss. The CDKN2A copy number loss group expressed significantly lower levels of CDKN2A mRNA and p16 protein than did the non-copy number loss group. Median OS for patients with and without CDKN2A copy number loss was 16.5 and 46.6 months, respectively (*p* = 0.007). Median DFS for both groups was 11.6 and 19.2 months, respectively (*p* = 0.03). In both univariate and multivariable analyses, stage IV designation, receipt of chemotherapy and CDKN2A copy number loss were predictive of OS.

**Conclusion:**

CDKN2A copy number loss predicted poor survival independently of other patient and treatment factors and may be a clinically useful prognostic factor.

## Introduction

It is well known that head and neck squamous cell carcinomas (HNSCCs) caused by human papillomavirus infection (HPV-positive) have considerably better prognosis than those not associated with HPV infection (HPV-negative) (1–3). To differentiate between HPV-positive and HPV-negative disease, p16 immunohistochemistry has historically been used; HPV viral protein E7 has been observed to downregulate pRb and subsequently increase p16 expression (4, 5). While the gold standard of HPV detection in head and neck cancers is now RNA-based detection of viral proteins E6/E7 (6), clinical and molecular analysis of p16 tumor data suggests that p16 may play an important role in the pathogenesis of head and neck cancers (7–10).

Given current understanding that HPV-positive and HPV-negative HNSCCs are clinically and biologically distinct, analysis of HNSCCs should ideally be stratified by HPV status. Interestingly, recent studies suggest that p16 expression varies greatly even among only HPV-positive or only HPV-negative HNSCCs (11–13). This wide variability in gene expression amongst patients with the same HPV status suggests that differences in p16 expression cannot be explained solely by HPV infection. CDKN2A, the gene that encodes p16, is frequently inactivated *via* copy number loss among HPV-negative head and neck cancer patients (14). Given the high prevalence of CDKN2A copy number loss, it is possible that this genomic abnormality may largely explain the wide variability in p16 expression among HPV-negative tumors. Moreover, considering the role of p16 as a known tumor suppressor, it is possible CDKN2A copy number loss may independently predict survival even when considering HPV-negative HNSCCs alone. We aim to investigate the emerging clinical significance of CDKN2A copy number loss in HPV-negative head and neck cancers using The Cancer Genome Atlas (TCGA).

## Materials and Methods

### Data Source and Study Population

The Cancer Genome Atlas is a joint effort by the National Cancer Institute and National Human Genome Research Institute that collected genomic and clinical patient data for 33 types of cancer. We analyzed TCGA head and neck cancer data, integrating various types of genomic measurements with clinical metadata. The TCGA data were analyzed as follows: previously published results of PCR-based RNA-detection of HPV E6/E7 RNA were used to identify HPV-negative cancers (15), and Affymetrix SNP6 copy number measurements were used to identify patients with CDKN2A copy number loss. CDKN2A mRNA expression (RNA-Seq v2) and p16 protein quantification (reverse phase protein array) were evaluated to characterize tumor CDKN2A expression. Since CDKN2A mRNA and p16 protein are downstream products of tumor CDKN2A DNA, mRNA and protein expression were expected to be relatively lower in individuals with CDKN2A copy number loss as long as the gene was transcriptionally and translationally active.

Inclusion criteria included patients with a primary tumor of known HPV-negative status in the oral cavity, oropharynx, hypopharynx, or larynx. Patients 80 years of age or greater were excluded for overall and disease-free survival (DFS) analyses. Clinical data were obtained from the TCGA Genomic Data Commons (16). Raw copy number data were acquired from the Broad Institute’s Genome Data Analysis Centers Firehose website (17). HPV status designations for this cohort were downloaded from the supplementary files of a recent publication by Nulton et al. (15) mRNA counts and protein expression data were obtained from the MSKCC Cancer Genomics Data Server through the “cgdsr” R package (18).

### Statistical Analyses and Variable Definitions

Copy number loss is defined as the loss of a chromosomal segment (e.g., during DNA replication). Loss of one or both copies of a gene contained in the deleted chromosomal segment often results in functional deficit due to gene under-expression (19). The primary independent variable, CDKN2A copy number loss, was defined *a priori* as having a relative log_2_ copy number ratio <−1. Kolmogorov–Smirnov testing was performed to evaluate differences in mRNA and protein expression between groups, both to validate CDKN2A copy number loss status and to investigate the transcriptional and translational effects of copy number loss. mRNA read count data were preprocessed by library size normalization using the TMM method, followed by log-transformation and *z*-scoring of mRNA reads (20).

Overall survival (OS) and DFS were the outcome variables examined. Wilcoxon rank-sum and χ^2^ tests were performed to assess the relationships between CDKN2A copy number loss and various demographic, clinicopathologic, and treatment variables. Survival analyses were conducted using the Kaplan–Meier method with log-rank testing for significance. Cox proportional hazards models with and without multiple imputation of missing values were also fit to identify demographic, clinicopathologic, and treatment factors associated with survival. Feature selection for multivariable analyses was performed by including clinicopathologic features previously reported to be prognostic in HNSCCs, patient and treatment factors found to be prognostic in our univariate Cox regressions, and clinicopathologic variables that differed significantly in prevalence between our copy number loss and non-copy number loss groups. All independence and hypothesis tests were performed using a two-sided significance level of 0.05. R version 3.4.1 and the following R packages were used to perform all data visualization and statistical analyses: “ggplot2,” “survival,” “survminer,” “interval,” and “mice” (21–26).

## Results

### Demographic and Clinicopathologic Differences Between Genomic Groups

We identified 401 patients under age 80 with HPV-negative head and neck cancer. Of these 401 patients, 146 (36.4%) exhibited CDKN2A copy number loss. The median age of all HPV-negative patients was 61 and the range was 19–79. The cohort tended to be mostly male (73.1%), with no significant difference in sex distribution between copy number groups. Anatomic site of lesion varied: 223 (56%) were cancers of the oral cavity, 66 (16%) were of the oropharynx, 105 (26%) were of the larynx, and 6 (1%) were of the hypopharynx. Clinical stage was used in place of pathologic stage for 44 patients for whom clinical stage but not pathologic stage information was available. The CDKN2A copy number loss group consisted of a slightly greater proportion of African Americans and Stage III/IV tumors and tended to have higher rates of smoking and heavy alcohol consumption (Table 1). The only statistically significant differences between groups at a 0.05 significance level were with regards to smoking status and heavy alcohol consumption.

**Table 1:** Demographic and clinicopathologic characteristics of all HPV-negative head and neck squamous cell carcinomas stratified by CDKN2A copy number.

Characteristic	CDKN2A copy num. loss (*N* = 146)	No CDKN2A copy num. loss (*N* = 255)	*p* Value
Age—mean (SD)	60.0 (9.7)	59.9 (11.7)	0.64
Race—no. (%)			0.10
White	117 (80.1)	215 (84.3)	
Black or African American	22 (15.1)	20 (7.8)	
Asian	4 (2.7)	6 (2.4)	
Native American	0 (0)	2 (0.8)	
Unknown	3 (2.1)	12 (4.7)	
Sex—no. (%)			0.28
Male	101 (69.2)	192 (75.3)	
Female	45 (30.8)	62 (24.3)	
Unknown	0 (0)	1 (0.4)	
AJCC pathologic stage—no. (%)			0.19
I	5 (3.4)	15 (5.9)	
II	20 (13.7)	42 (16.5)	
III	22 (15.1)	54 (21.2)	
IV	99 (67.8)	143 (56.1)	
Unknown	0 (0)	1 (0.4)	
Tumor location—no. (%)			0.78
Oral cavity	77 (52.7)	146 (57.3)	
Oropharynx	26 (17.8)	40 (15.7)	
Larynx	40 (27.4)	65 (25.5)	
Hypopharynx	3 (2.1)	3 (1.2)	
Unknown	0 (0)	1 (0.4)	
Tobacco use—no. (%)			<0.03*
Never	21 (14.4)	69 (23.5)	
Current	63 (43.2)	87 (34.1)	
Former	58 (39.7)	104 (40.8)	
Unknown	4 (2.7)	4 (1.6)	
Tobacco pack years among ever smokers—mean (SD)	46.0 (29.3)	48.6 (40.7)	0.81
Alcohol consumption—no. (%)			<0.04*
Moderate or non-drinker	21 (14.4)	65 (25.5)	
Heavy drinker^a^	31 (21.2)	48 (18.8)	
Unknown	94 (64.4)	142 (55.7)	
Chemotherapy—no. (%)			0.60
Yes	53 (36.3)	80 (31.4)	
No	33 (22.6)	62 (24.3)	
Unknown	60 (41.1)	113 (44.3)	
Radiation—no. (%)			0.80
Yes	85 (58.2)	140 (54.9)	
No	38 (26.0)	73 (28.6)	
Unknown	23 (15.8)	42 (16.5)	

*^a^Defined as >14 drinks/week or >4 drinks/day for men or >7 drinks/week or >3 drinks/day for women*.

*p < 0.05, statistically significant.

### mRNA/Protein Expression Differences Between Test and Control Groups

**Figure 1:**
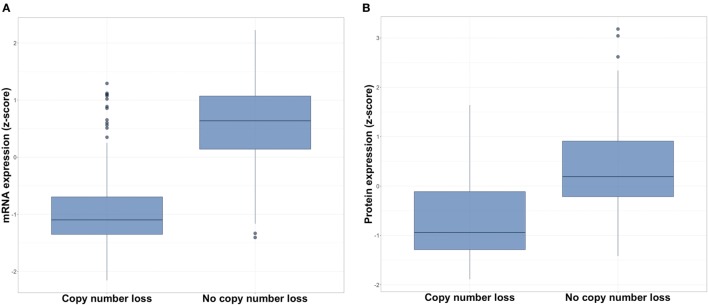
Expression of **(A)** CDKN2A mRNA and **(B)** p16 protein in copy number loss vs. non-copy number loss groups for all HPV-negative head and neck squamous cell carcinomas.

The CDKN2A copy number loss group exhibited significantly lower CDKN2A mRNA expression than did the non-copy number loss group (median −1.10 vs. 0.64, *p* < 2.2 × 10^−16^; Figure 1A). Similarly, analysis of protein assay results for 174 samples for which RPPA data were available revealed a lower expression of p16 in the CDKN2A copy number loss group (median −0.94 vs. 0.19, *p* < 7.1 × 10^−11^; Figure 1B). These results corroborate CDKN2A copy number loss status assignments. Furthermore, they demonstrate that CDKN2A copy number loss has a functional impact on gene transcription and translation.

### Survival Analysis

**Figure 2:**
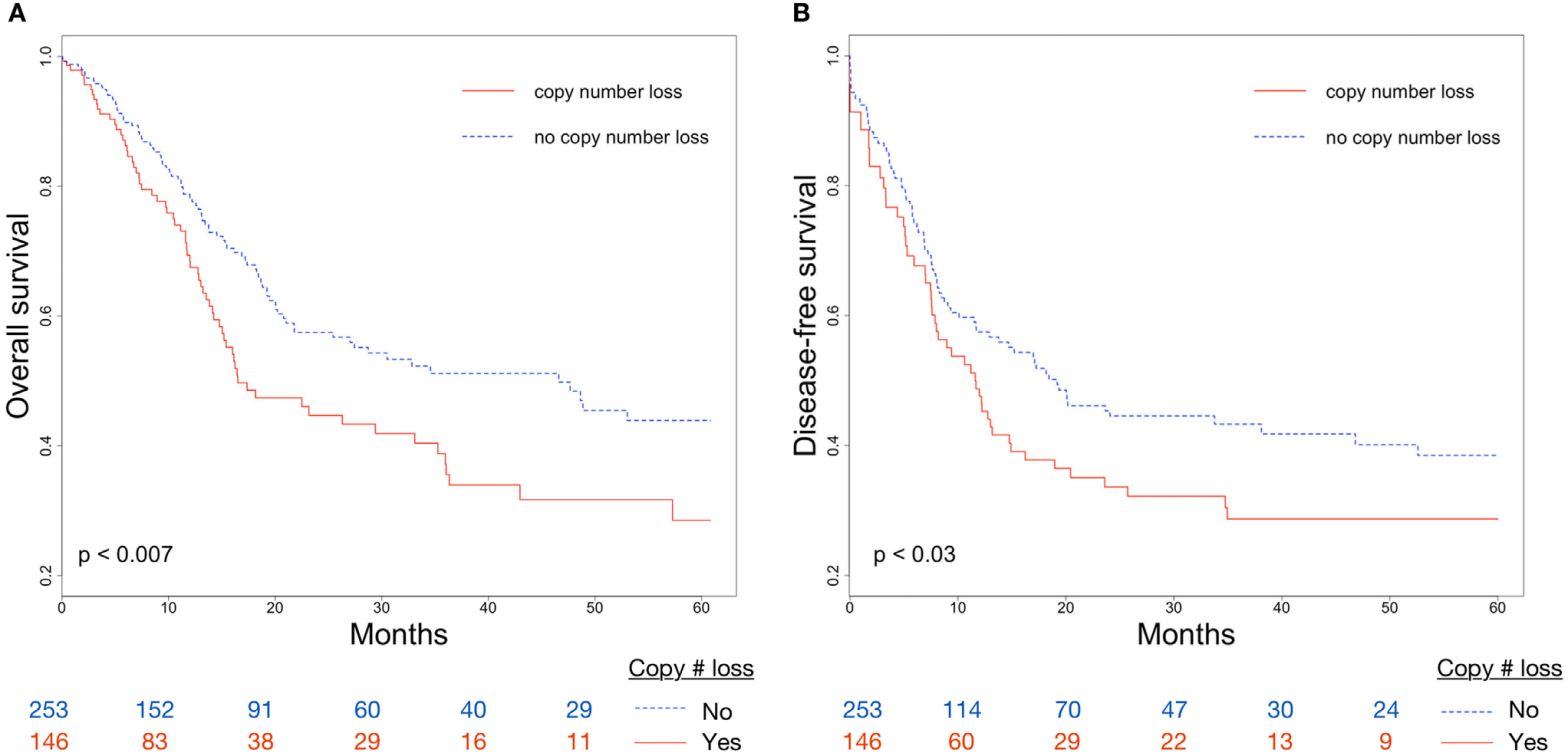
Differences in **(A)** overall survival (OS) and **(B)** disease-free survival (DFS) between CDKN2A copy number loss and non-copy number loss groups for all tumors.

**Figure 3:**
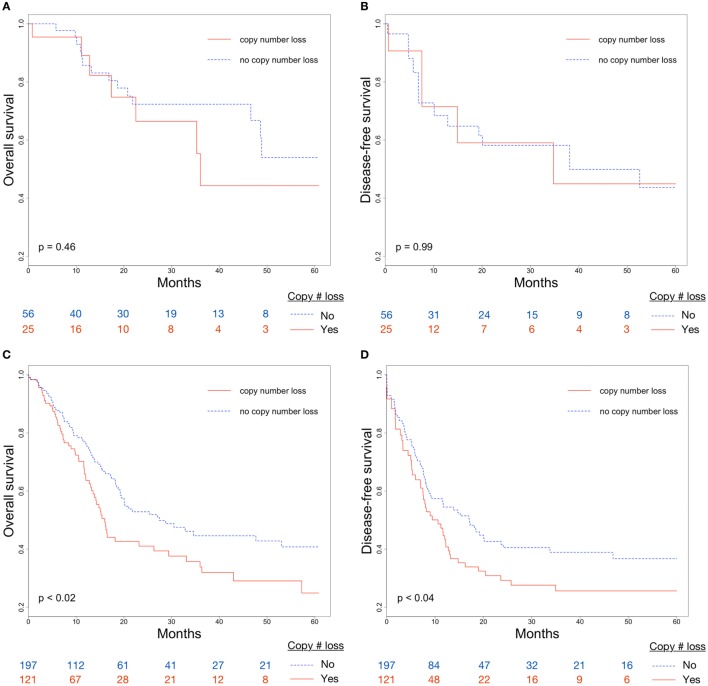
Differences in **(A)** overall survival (OS) and **(B)** disease-free survival (DFS) between CDKN2A copy number loss and non-copy number loss groups for early-stage (stage I/II) tumors; Differences in **(C)** OS and **(D)** DFS between CDKN2A copy number loss and non-copy number loss groups for advanced-stage (stage III/IV) tumors.

Overall survival and DFS were examined to evaluate the clinical implications of CDKN2A copy number loss. Median OS for patients with and without CDKN2A copy number loss were 16.5 and 46.6 months, respectively (*p* < 0.007; Figure 2A). CDKN2A copy number loss was also significantly associated with decreased DFS (median 11.6 vs. 19.2 months, *p* < 0.03; Figure 2B). There was no significant difference in OS or DFS between groups for early-stage (stage I or II) disease (Figures 3A,B), but among advanced-stage (stage III or IV) tumors, copy number loss was indicative of poor OS (median 16.0 vs. 27.4 months, *p* < 0.02; Figure 3C) and DFS (median 9.0 vs. 17.0 months, *p* < 0.04; Figure 3D).

On univariate Cox proportional hazards analysis, African American race (HR: 1.70; 95% CI: 1.05–2.76), Stage IV AJCC pathologic tumor stage (HR: 3.19; 95% CI: 1.01–10.03), receipt of chemotherapy (HR: 1.68; 95% CI: 1.22–2.33), and CDKN2A copy number loss (HR: 1.54; 95% CI: 1.12–2.11) were associated with poor OS (Table 2). In adjusted analysis using a multivariable Cox proportional hazards model, AJCC pathologic tumor stage (HR: 1.86; 95% CI: 1.06–3.26), receipt of chemotherapy (HR: 1.87; 95% CI: 1.12–3.12), and CDKN2A copy number loss (HR: 1.42; 95% CI: 1.01–2.00) remained significant (Table 3). Given the considerable amount of unrecorded data on alcohol consumption and receipt of chemotherapy, multiple imputation was performed and multivariable Cox regression was applied to the imputed dataset. This analysis revealed that the same variables of tumor stage (HR: 1.79, 95% CI: 1.03–3.11), receipt of chemotherapy (HR: 1.64, 95% CI: 1.13–2.37), and CDKN2A copy number loss (HR: 1.51, 95% CI: 1.06–2.16) were independently associated with poor survival (Table 3).

**Table 2:** Univariable Cox proportional hazards analysis of all HPV-negative head and neck squamous cell carcinomas.

Variable (ref.)	Hazard ratio	95% CI	*p* Value
Age (<50)	Ref.	Ref.	Ref.
50–64	0.72	0.47–1.11	0.14
65–79	0.86	0.55–1.33	0.50
Race (white)	Ref.	Ref.	Ref.
Black or African American	1.70	1.05–2.76	<0.04*
Asian	1.22	0.45–3.29	0.70
Native American	3e−7	–	Inf.
Sex (male)	Ref.	Ref.	Ref.
Female	1.12	0.80–1.58	0.51
AJCC pathologic stage (I)	Ref.	Ref.	Ref.
II	1.50	0.44–5.10	0.51
III	2.20	0.67–7.29	0.19
IV	3.19	1.01–10.03	<0.05*
Tumor location (oral cavity)	Ref.	Ref.	Ref.
Oropharynx	0.87	0.57–1.32	0.51
Larynx	0.86	0.59–1.26	0.44
Hypopharynx	1.07	0.26–4.34	0.93
Tobacco use (never)	Ref.	Ref.	Ref.
Current	1.52	0.98–2.36	0.06
Former	0.93	0.60–1.45	0.75
Alcohol consumption (none/moderate)	Ref.	Ref.	Ref.
Heavy	1.33	0.79–2.24	0.29
Chemotherapy (no)			
Yes	1.68	1.22–2.33	<0.002*
Radiation (no)	Ref.	Ref.	Ref.
Yes	0.71	0.50–1.02	0.06
CDKN2A (no copy # loss)			
Copy number loss	1.54	1.12–2.11	<0.008*

*p < 0.05, statistically significant.

**Table 3:** Multivariable Cox proportional hazards model of all HPV-negative head and neck squamous cell carcinomas.

Variable (ref.)	No imputation			Multiple imputation		
	Hazard ratio	95% CI	*p*Value	Hazard ratio	95% CI	*p* Value
Age (<50)	Ref.	Ref.	Ref.	Ref.	Ref.	Ref.
50–64	0.67	0.43–1.06	0.09	0.72	0.46–1.13	0.15
65–79	1.02	0.62–1.67	0.93	1.08	0.66–1.77	0.77
Race (white)	Ref.	Ref.	Ref.	Ref.	Ref.	Ref.
Black or African American	1.52	0.90–2.57	0.12	1.56	0.92–2.64	0.10
Asian	1.31	0.46–3.75	0.99	1.33	0.47–3.74	0.60
Native American	5e−7	–	Inf.	3e−7	–	Inf.
Sex (male)	Ref.	Ref.	Ref.	Ref.	Ref.	Ref.
Female	1.11	0.76–1.61	0.60	1.11	0.76–1.62	0.58
AJCC Pathologic Stage (II)^a^	Ref.	Ref.	Ref.	Ref.	Ref.	Ref.
I	0.91	0.26–3.15	0.88	0.93	0.27–3.24	0.91
III	1.55	0.82–2.94	0.18	1.55	0.82–2.93	0.18
IV	1.86	1.06–3.26	<0.04*	1.79	1.03–3.11	<0.04*
Smoking status (never)	Ref.	Ref.	Ref.	Ref.	Ref.	Ref.
Current	1.42	0.86–2.34	0.17	1.41	0.86–2.30	0.17
Former	0.97	0.59–1.60	0.91	1.00	0.61–1.63	0.99
Heavy alcohol use (no)	Ref.	Ref.	Ref.	Ref.	Ref.	Ref.
Yes	1.14	0.66–1.97	0.64	0.81	0.57–1.16	0.25
Unknown	1.02	0.64–1.62	0.94	–	–	–
Receipt of chemotherapy (no)	Ref.	Ref.	Ref.	Ref.	Ref.	Ref.
Yes	1.87	1.12–3.12	<0.02*	1.64	1.13–2.37	<0.01*
Unknown	1.49	0.91–2.45	0.11	–	–	–
CDKN2A (no copy num. loss)	Ref.	Ref.	Ref.	Ref.	Ref.	Ref.
Copy number loss	1.42	1.01–2.00	<0.05*	1.51	1.06–2.16	<0.03*

*^a^Stage II used as reference group because few Stage I tumors*.

*p < 0.05, statistically significant.

## Discussion

In our integrated genomic and clinical analysis of TCGA, CDKN2A copy number loss was associated with poor prognosis in HPV-negative head and neck cancer independently of other known prognostic factors including age, advanced tumor stage, and African American race (13, 14, 27). CDKN2A copy number loss was also strongly associated with decreased CDKN2A mRNA and protein expression, demonstrating significant impact on gene transcription and translation.

Univariate survival analysis found CDKN2A copy number loss to indicate worse prognosis in all HPV-negative disease. Stratifying by copy number and sub-stratifying by stage (early vs. late) showed that CDKN2A copy number loss indicated significantly poorer OS and DFS in advanced-stage but not early-stage disease. Given that our cohort consists primarily of advanced-stage tumors, lack of observed survival difference in the early-stage cohort may be due to low sample size. Follow-up studies with a greater number of early-stage tumors would be useful to validate this finding.

Notably, copy number loss retained prognostic value on multivariable analysis. For this analysis, we included possible covariates identified by reviewing previous reports of patient factors predictive of survival and conducting univariate Cox regressions on our own data. Additionally, we included the variables of smoking and heavy alcohol consumption (which were both found to have significantly different prevalence between compared groups) in our multivariable Cox regression to identify possible confounders. The finding that high pathologic stage is an independent predictor of survival is consistent with previous findings, and the finding that receipt of chemotherapy also independently predicts poor survival is not too surprising considering that patients who receive chemotherapy tend to have more advanced, systemic disease even amongst high-stage cancers. The finding that African American race was predictive of survival on univariate analysis has precedence (13), and its prognostic value was not maintained in our multivariate analysis. This too is consistent with previous reports suggesting that the difference in survival between racial groups is likely related to socioeconomic factors resulting in treatment disparities (28). Interestingly, CDKN2A copy number loss was found to be significant on multivariable analysis, suggesting it may be clinically useful as an independent prognostic factor.

Some missing or unavailable clinical data limit the conclusions that can be drawn from this study. For instance, data on receipt of chemotherapy and adjuvant radiotherapy were sparse (missing in 43 and 16% of patients, respectively). These variables are of particular clinical interest, as outcomes analysis of individuals who receive these treatments can reveal insights into efficacy and help shape best practice guidelines. We were able to incorporate variables with sparse data into our multivariable analyses through categorical representation of unknowns or through multiple imputation, but these approaches are not perfect substitutes for actual values. Thus, as highlighted in a recent editorial, we stress the importance of documenting such treatment information more completely in future data collection efforts (29). Another limitation encountered was the absence of comorbidity data. We had access to many major demographic and clinicopathologic variables, but full comorbidity histories were not available. Such data are helpful when performing retrospective cohort-based analyses to more comprehensively control for clinical confounders. Additionally, though we used overall and DFS as outcome measures, cancer-specific survival would have been ideal. To facilitate access to comorbidity and cancer-specific survival data and to provide researchers with more complete longitudinal clinical data, we suggest that future genomics data collection efforts like TCGA consider linking with Medicare to provide researchers with more complete longitudinal clinical data for patients age 65 and older.

Despite these limitations, this study highlights strengths of the dataset and our integrated approach to clinical and genomic analysis. To our knowledge, of all clinically oriented genomic studies of HNSCCs, this cohort includes the largest collection of HPV-negative HNSCCs to date. In genomics research, sample size is often limited because of the extensive costs and workflow required to acquire and sequence patient samples (30). With the large number of samples in TCGA, we believe that our cohort is more representative of the total population of HPV-negative HNSCCs than smaller HNSCC cohorts of previously published clinical analyses. A methodologic strength of this study is that it integrates data from different sequencing platforms to validate the functional significance of the genomic abnormality, prior to survival analysis. We emphasize the importance of including complementary mRNA and protein expression data when evaluating mutations and copy number alterations, as not all mutations or abnormalities of a given gene have the same (or any) effect. Transcriptional and translational analysis can provide insights into the biological relevance of mutations and other genomic abnormalities in the context of other disease influences.

In conclusion, we found that CDKN2A copy number loss was associated with low expression of CDKN2A mRNA and p16 protein and indicated poor clinical prognosis in terms of disease progression and OS. These survival differences remained significant on multivariable analysis, suggesting CDKN2A copy number loss may have clinical utility as an independent prognostic factor for advanced-stage HNSCC. Through this analysis, we demonstrate the power and limitations of the TCGA database in analyzing the clinical impact of a genomic abnormality. Future large-scale genomic data collection efforts should emphasize linking genomic data with robust, longitudinal treatment and outcomes data to accelerate clinical discovery.

## Author Contributions

WC and JY conceived of the presented idea. WC, RB, ZH, JC, and JY contributed to experimental design, selection of outcomes measures, and variable selection. WC preprocessed and performed the initial data analysis. WC, RB, AM, TH, ZH, JC, and JY contributed to interpretation of the results. SG and JT verified the analytical methods. JY supervised the findings of this work. All authors discussed the results and contributed to writing the final manuscript.

## Conflict of Interest Statement

JY receives research funding from 21st Century Oncology. All other authors declare that the research was conducted in the absence of any commercial or financial relationships that could be construed as a potential conflict of interest.
